# Phthalide derivative CD21 attenuates tissue plasminogen activator-induced hemorrhagic transformation in ischemic stroke by enhancing macrophage scavenger receptor 1-mediated DAMP (peroxiredoxin 1) clearance

**DOI:** 10.1186/s12974-021-02170-7

**Published:** 2021-06-24

**Authors:** Dong-Ling Liu, Zhi Hong, Jing-Ying Li, Yu-Xin Yang, Chu Chen, Jun-Rong Du

**Affiliations:** 1 Department of Pharmacology, West China School of Pharmacy, Sichuan University, Chengdu, 610041 PR China; 2 Present address: The PRIVIS TECHNOLOGY Co., Ltd., Chengdu, 610041 PR China; 3 Sichuan Academy of Chinese Medicine Sciences, Chengdu, 610041 PR China

**Keywords:** Ischemic stroke, Tissue plasminogen activator, Hemorrhagic transformation, DAMP (peroxiredoxin 1) clearance, MSR1, TLR4/NF-κB pathway, Neuroinflammation

## Abstract

**Background:**

Hemorrhagic transformation (HT) is a critical issue in thrombolytic therapy in acute ischemic stroke. Damage-associated molecular pattern (DAMP)-stimulated sterile neuroinflammation plays a crucial role in the development of thrombolysis-associated HT. Our previous study showed that the phthalide derivative CD21 attenuated neuroinflammation and brain injury in rodent models of ischemic stroke. The present study explored the effects and underlying mechanism of action of CD21 on tissue plasminogen activator (tPA)-induced HT in a mouse model of transient middle cerebral artery occlusion (tMCAO) and cultured primary microglial cells.

**Methods:**

The tMCAO model was induced by 2 h occlusion of the left middle cerebral artery with polylysine-coated sutures in wildtype (WT) mice and macrophage scavenger receptor 1 knockout (MSR1^−/−^) mice. At the onset of reperfusion, tPA (10 mg/kg) was intravenously administered within 30 min, followed by an intravenous injection of CD21 (13.79 mg/kg/day). Neuropathological changes were detected in mice 3 days after surgery. The effect of CD21 on phagocytosis of the DAMP peroxiredoxin 1 (Prx1) in lysosomes was observed in cultured primary microglial cells from brain tissues of WT and MSR1^−/−^ mice.

**Results:**

Seventy-two hours after brain ischemia, CD21 significantly attenuated neurobehavioral dysfunction and infarct volume. The tPA-infused group exhibited more severe brain dysfunction and hemorrhage. Compared with tPA alone, combined treatment with tPA and CD21 significantly attenuated ischemic brain injury and hemorrhage. Combined treatment significantly decreased Evans blue extravasation, matrix metalloproteinase 9 expression and activity, extracellular Prx1 content, proinflammatory cytokine mRNA levels, glial cells, and Toll-like receptor 4 (TLR4)/nuclear factor κB (NF-κB) pathway activation and increased the expression of tight junction proteins (zonula occludens-1 and claudin-5), V-maf musculoaponeurotic fibrosarcoma oncogene homolog B, and MSR1. MSR1 knockout significantly abolished the protective effect of CD21 against tPA-induced HT in tMCAO mice. Moreover, the CD21-induced phagocytosis of Prx1 was MSR1-dependent in cultured primary microglial cells from WT and MSR1^−/−^ mice, respectively.

**Conclusion:**

The phthalide derivative CD21 attenuated tPA-induced HT in acute ischemic stroke by promoting MSR1-induced DAMP (Prx1) clearance and inhibition of the TLR4/NF-κB pathway and neuroinflammation.

**Supplementary Information:**

The online version contains supplementary material available at 10.1186/s12974-021-02170-7.

## Background

Stroke is the leading cause of morbidity and mortality in adults worldwide. Ischemic stroke accounts for > 80% of all stroke cases [1]. Cerebral hypoperfusion and oxygen/energy shortage are the main causes of brain injury in ischemic stroke. Early thrombolysis with tissue plasminogen activator (tPA) to restore blood supply to the ischemic area has been widely used for the clinical treatment of acute ischemic stroke [2]. This revascularization therapy, however, has a narrow therapeutic time window (3–4.5 h), which greatly limits its clinical utility [3]. Moreover, many patients suffer from severe side effects, particularly hemorrhagic transformation (HT), after thrombolysis with tPA [2, 3]. This cerebral HT results in a worse clinical prognosis and even death in ischemic stroke patients who receive tPA treatment. Therefore, strategies that can ameliorate tPA-related HT would significantly improve clinical thrombolytic treatment outcomes.

Numerous studies have demonstrated that sterile inflammation aggravates brain injury and neurological dysfunction in both ischemic and hemorrhagic stroke [4–6]. Moreover, neuroinflammation has been found to strongly contribute to the development of blood-brain barrier (BBB) disruption and subsequent HT that are caused by thrombolysis after acute ischemic stroke [7, 8]. A recent study reported that an essential mechanism of tPA-induced HT involves the Toll-like receptor 4 (TLR4) signaling pathway [8]. Once activated by damage-associated molecular patterns (DAMPs), TLR4 may induce the nuclear translocation of nuclear factor κB (NF-κB) p65 and trigger NF-κB-dependent transcriptional activity, thereby promoting the production of a range of inflammatory mediators (e.g., cytokines, chemokines, and matrix metalloproteinases [MMPs]) and exacerbating post-ischemic brain damage and HT [8–10]. Studies have gradually identified post-stroke DAMPs, such as high-mobility-group box 1 (HMGB1) and peroxiredoxins (Prxs) [6, 10]. Peroxiredoxins comprise a family of highly conserved antioxidant enzymes that are more abundant in the brain than in other tissues and can be either passively released from necrotic cells or actively secreted from wounded cells, resulting in TLR4/NF-κB pathway activation and a strong inflammatory response [11, 12]. Studies by our group and others have demonstrated the proinflammatory properties of extracellular Prxs in experimental stroke. The extracellular increase in Prxs may function as DAMPs to interact with TLR4, leading to NF-κB-dependent neuroinflammatory responses in rodent models of ischemic and hemorrhagic stroke [11, 13–16]. In contrast to HMGB1 that is rapidly released during the hyperacute phase of brain ischemia (i.e., within 6 h after stroke onset), ischemic stress-induced Prxs are extracellularly released during the acute phase of ischemia (i.e., 12–24 h after stroke onset) [17]. Therefore, Prx-based therapy is a potential strategy with a wide therapeutic window for post-stroke inflammation. Interestingly, emerging evidence suggests that DAMPs are internalized and cleared in the lysosome in a macrophage scavenger receptor 1 (MSR1)-dependent manner [18, 19]. Neuroinflammation that is stimulated by DAMPs (e.g., HMGB1 and Prxs) after brain ischemia was shown to be negatively regulated by MSR1, which conferred potent neuroprotection within a 24-h therapeutic time window in mice that were subjected to transient middle cerebral artery occlusion (tMCAO) [18]. Moreover, MSR1 promoted macrophage M2-like polarization and induced the osteogenic differentiation of bone marrow stem cells (BMSCs) in a co-culture system [20]. Notably, recent research found that plasma levels of HMGB1 significantly increased in tPA-treated ischemic stroke patients, and HMGB1-triggered neuroinflammation aggravated tPA-mediated HT in a rat model of ischemic stroke [21, 22]. Nevertheless, the therapeutic potential of MSR1 for thrombolysis-induced HT after acute ischemic stroke has not yet been reported.

Numerous experimental and clinical studies have reported protective effects of natural phthalides against ischemic stroke [14, 23, 24]. Butylphthalide was the first natural phthalide that was approved in China for the treatment of acute ischemic stroke. Combined treatment with tPA and butylphthalide was recently reported to reduce the incidence of HT and improve prognosis in cerebral ischemic patients, and these beneficial effects were related to the alleviation of tPA-induced neuroinflammation [24]. CD21 is a patented, newly designed phthalide derivative that is synthesized based on natural phthalides (Supplementary Figure S1) [25]. Our previous study found that CD21 dose-dependently and significantly ameliorated neuroinflammation and brain injury in rodent models of ischemic stroke. Its neuroprotective mechanism of action was associated with the induction of microglial/macrophage M2 polarization and MSR1-promoted DAMP (Prx1)/TLR4/NF-κB pathway inhibition [19, 26, 27]. Based on these findings, the present study further explored the effects and underlying mechanism of action of CD21 on tPA-induced neurovascular complications in acute ischemic stroke. The present findings demonstrated the involvement of the DAMP Prx1 in tPA-induced BBB dysfunction and HT after brain ischemia, which were significantly ameliorated by CD21 through the upregulation of MSR1-promoted DAMP (Prx1) clearance and inhibition of TLR4/NF-κB in tMCAO mice and cultured primary microglial cells.

## Methods

### Reagents

The antibodies that were used in the study are listed in Table 1. The specific primer pairs for polymerase chain reaction (PCR, Beijing Genomics Institute, Beijing, China) are shown in Table 2. tPA was purchased from Boehringer Ingelheim (catalog no. S20160055, Ingelheim, Germany). Prx1 protein was obtained from Sino Biological (Beijing, China) and conjugated with AbFluor 488 dyes (Abbkine, Wuhan, China) according to the manufacturer’s protocol. Trizol reagent and LysoTracker were purchased from Invitrogen Life Technologies (Carlsbad, CA, USA), and 2,3,5-triphenyltetrazoliumchloride (TTC) was obtained from Sigma-Aldrich (St. Louis, MO, USA). The Gelatin-Zymography Kit and BCA protein assay kit were obtained from Xinfan and Beyotime (Shanghai, China), respectively. The nuclear protein extraction kit was purchased from Boster (Wuhan, China). The DeadEnd Fluorometric TUNEL System was obtained from Promega (Madison, WI, USA). All of the other reagents were obtained from local commercial sources.

**Table 1:** Primary antibodies used in this study

Antibody	Dilution	Application	Source
CD206	1:50	Immunofluorescence	Abcam, USA
CD31	1:20	Immunofluorescence	Abcam, USA
CD86	1:50	Immunofluorescence	Abcam, USA
Claudin-5	1:300	Western blot	Wanleibio, China
GFAP	1:100	Immunofluorescence	Wanleibio, China
Histone H3	1:500	Western blot	Wanleibio, China
IBA-1	1:500	Immunofluorescence	Wako, Japan
MAFB	1:200	Western blot	Bioss, China
MMP9	1:50	Immunofluorescence	Abcam, USA
MSR1	1:200	Western blot	Bioss, China
	1:100	Immunofluorescence	
MyD88	1:200	Western blot	Bioss, China
NF-κB p65	1:500	Western blot	Bioss, China
Pan-cadherin	1:100	Immunofluorescence	Abcam, USA
Prx1	1:100 1:1000	Immunofluorescence, Western blot	Abcam, USA
TLR4	1:500	Western blot	Signalway Antibody Biosciences, USA
ZO-1	1:500	Western blot	Wanleibio, China
β-actin	1:2000	Western blot	ZSGB-Bio, China

**Table 2:** Specific primer pairs used for polymerase chain reaction

Gene	Forward	Reverse
TNF-α	5′-TCAGCCTCTTCTCATTCCTGC-3′	5′-TTGGTGGTTTGCTACGACGTG-3′
IL-1β	5′-CAGCAATGGTCGGGAC-3′	5′-TAGGTAAGTGGTTGCCT-3′
IL-6	5′-CAGCAATGGTCGGGAC-3′	5′-TAGGTAAGTGGTTGCCT-3′
GAPDH	5′-AGCAGTCCCGTACACTGGCAAAC-3′	5′-′TCTGTGGTGATGTAAATGTCCTCT-3′

### Animals and induction of focal brain ischemia

Adult male C57BL/6 wildtype (WT) mice, weighing 23–27 g, were purchased from Byrness Weil Biotech Ltd (Chongqing, China). MSR1 knockout (MSR1^−/−^) mice on a C57BL/6 background were provided by Prof. Qi Chen [20]. The animals were housed under a 12 h/12 h light/dark cycle with controlled temperature and humidity. Food and water were freely available. All of the experiments were performed in accordance with the ethical guidelines of the Animal Ethics Committee of Sichuan University.

The mice were anesthetized via the inhalation of 2% isoflurane. The tMCAO model was induced by inserting a polylysine-coated suture (Beijing Cinontech, Beijing, China) into the origin of the left middle cerebral artery (MCA) through the external carotid artery (ECA) and internal carotid artery (ICA). Two hours later, the suture was withdrawn, and the brain was reperfused [28]. The mice were allowed to breathe spontaneously. Rectal temperature was maintained at 37 °C throughout the procedure. Cerebral blood flow was monitored by laser Doppler flowmetry. Focal cerebral blood flow decreased approximately 75% after occlusion, and recovery after reperfusion demonstrated success of the mouse model of tMCAO [29]. Sham control mice underwent similar surgical procedures but without MCA blockade.

### Pharmacological treatments for ischemic stroke in mice

**Fig. 1:**
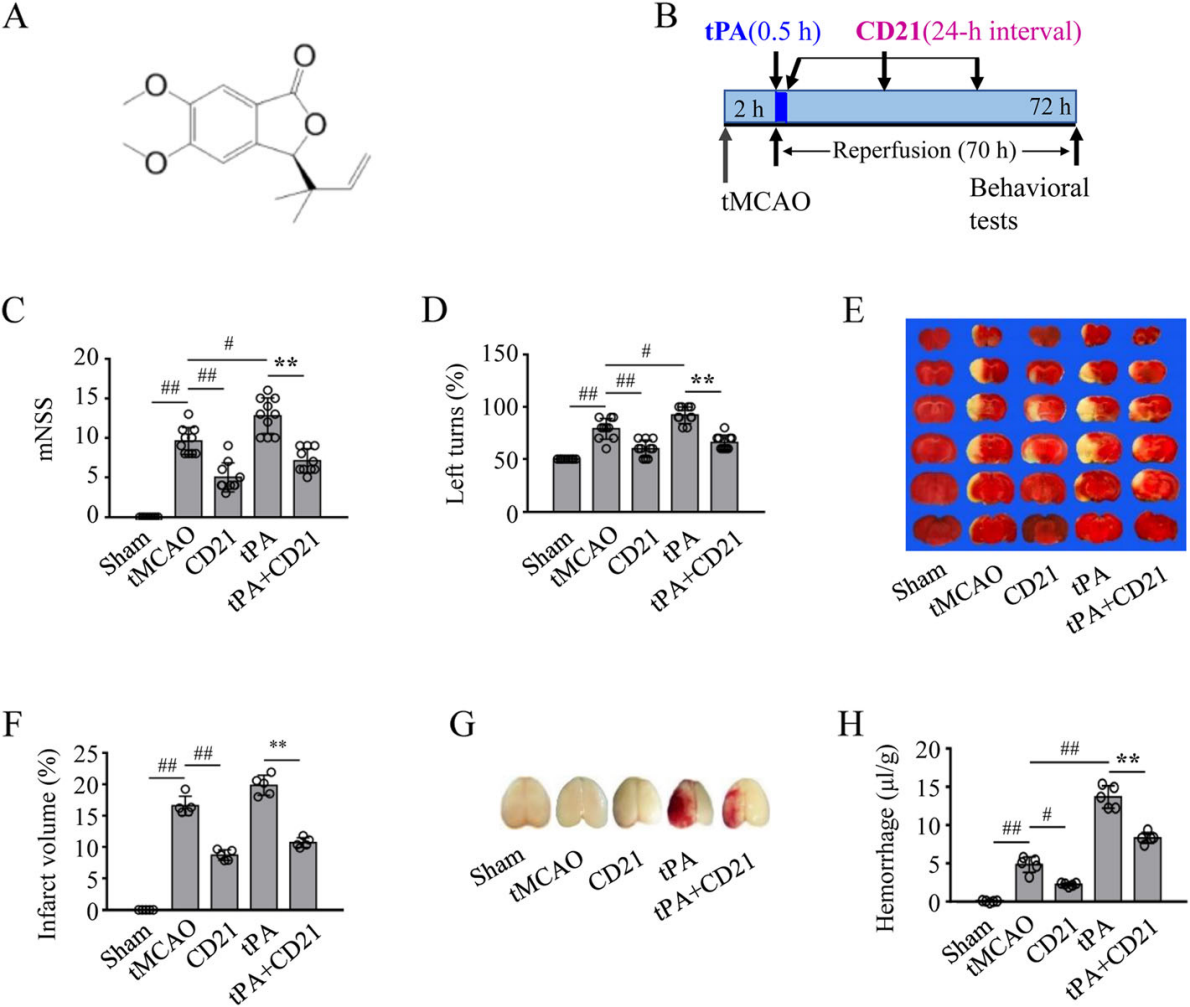
CD21 protected against ischemic brain injury and hemorrhagic transformation in tPA-infused tMCAO mice. **a** Chemical structure of CD21. **b** Overall experimental design. **c**, **d** mNSS and Corner test scores. **e**, **f** Representative images and statistical analysis of infarct volume by TTC staining. **g**, **h** Representative images of the hemorrhagic brain and statistical analysis of hemorrhagic volume in the ipsilateral brain hemisphere. The data are expressed as mean ± SD (*n* = 10/group in **c** and **d**, *n* = 5/group in **e**–**h**). ^#^*p* < 0.05, ^##^*p* < 0.01, vs. tMCAO group; **p < 0.01, vs. tPA group

Butylphthalide is an approved natural phthalide for the clinical treatment of ischemic stroke in China. The 10 mg/kg intravenous dose in mice per day is equivalent to the clinical treatment regimen in patients. The molecular weights of butylphthalide and CD21 are 190 and 262, respectively (Figure S1). Therefore, 13.79 mg/kg CD21 is an intravenously equimolar dose of 10 mg/kg butylphthalide in mice. CD21 (> 99% purity; Supplementary Figure S2) was formulated in saline with 10% anhydrous ethanol, 10% soybean oil, and 0.4% Tween-80. At the onset of reperfusion after 2-h suture occlusion in tMCAO mice, tPA (10 mg/kg) was administered via the tail vein within 30 min using a minipump, followed by an intravenous injection of 13.79 mg/kg CD21 once daily for 3 days (Fig. 1b) [30].

### Examination of neurological deficits

Seventy-two hours after ischemia, neurological impairment was evaluated in tMCAO mice using the modified neurological severity score (mNSS) and corner test. The mNSS is based on an 18-point scale: 0 (no significant deficits), 1–6 (mild neurobehavioral impairment), 7–12 (moderate neurobehavioral impairment), and 13–18 (severe neurobehavioral impairment) [31]. In the corner test, the percentage of left turns was calculated in 10 trials for each mouse. A higher percentage indicates more severe sensory dysfunction [32]. Behavioral testing was performed by researchers who were blind to the treatment groups.

### Examination of infarct volume

After the neurobehavioral tests, the brains of tMCAO mice were rapidly removed under anesthesia and frozen at − 20 °C. Six brain slices of the same thickness were stained with TTC. The infarct volume was measured as the percentage of infarct volume in six slices relative to the total brain volume for each mouse [14].

### Measurement of hemoglobin levels

After the neurobehavioral tests, tMCAO mice were perfused through the heart with ice-cold saline under anesthesia and euthanized. The ipsilateral cerebral hemisphere of each mouse was isolated and homogenized for the measurement of hemoglobin levels using Drabkin’s reagent as described previously [28].

### Assessment of Evans blue extravasation

To assess BBB integrity in tMCAO mice, Evans blue (4% in saline, 4 ml/kg) was intravenously injected 69 h after surgery. Three hours later, the mice were perfused through the heart with ice-cold saline under anesthesia, and the ipsilateral brain hemisphere was collected and homogenized with *N*,*N*-dimethylformamide, respectively. The extravasation of Evans blue was assessed according to the following formula: {*A*_620 nm_ – [(*A*_500 nm_ + *A*_740 nm_) / 2]} / mg wet weight [28].

### Measurement of matrix metalloproteinase 9 activity

The activity of cerebral metalloproteinase 9 (MMP-9) was measured using the Gelatin-Zymography Kit according to the manufacturer’s instructions. tMCAO mice were perfused with ice-cold saline under anesthesia 72 h after surgery. Total protein was extracted from the ipsilateral brain hemisphere, and the same amount of protein per mouse was electrophoresed in gelatin-containing sodium dodecyl sulfate-polyacrylamide gel electrophoresis gel. Coomassie brilliant blue staining was then performed after renaturation. Images were captured by a gel imager (Bio-Rad, Hercules, CA, USA), and transparent white stripes were analyzed using Gel Pro Analyzer 6.0 software (Media Cybernetics, Bethesda, MD, USA) [33].

### Immunofluorescent analysis

tMCAO mice were deeply anesthetized and perfused through the heart with ice-cold phosphate-buffered saline, followed by 4% paraformaldehyde 72 h after surgery. Brain tissues that encompassed the injury core were cut into 20 μm frozen sections for immunofluorescent staining after fixation and dehydration. A fluorescence microscope was used to observe the sections and acquire images, and the images were processed using ImagePro Plus 6.0 software [34].

To determine the expression and localization of Prx1 in the ischemic brain, the brain sections were first stained with the DeadEnd Fluorometric apoptosis detection system (Promega, Madison, WI, USA) for TUNEL staining according to the manufacturer’s instructions, followed by the immunofluorescent staining of Prx1 and pan-cadherin as described previously [18]. Photomicrographs were acquired under a fluorescence microscope with a × 40 objective (Nikon, Tokyo, Japan) and processed using ImageJ software (National Institutes of Health, Bethesda, MD, USA).

### Western blot

Seventy-two hours after surgery, ipsilateral brain hemispheres were collected from tMCAO mice. Total protein and nuclear protein were isolated from homogenates using RIPA buffer or a cytoplasmic and nuclear protein extraction kit, respectively. Equal amounts of proteins were analyzed by Western blot using primary antibodies (Table 1). The immunoblotting results were analyzed by a gel imager (Bio-Rad, California, USA) and are reported as the optical density relative to the sham group.

### Quantitative real-time polymerase chain reaction

Seventy-two hours after surgery, ipsilateral brain hemispheres were collected from tMCAO mice. Total RNA was isolated using Trizol reagent, and cDNA was obtained using a reverse transcription method. Quantitative real-time PCR (qPCR) was performed using a LightCycler96 PCR device (Roche, Basel, Switzerland) using specific primer pairs (Table 2) [14].

### Phagocytosis of Prx1 in lysosomes in cultured primary microglia

The brains of WT and MSR1^−/−^ mice were obtained within 24 h of birth, and meninges and blood vessels were removed. Brain tissue fragments were then digested with 0.25% trypsin and filtered to obtain a cell suspension. The resuspended sediment was cultured in DMED-F12 medium that contained 10% serum, and primary microglia were collected by shaking the upright flask after the apparent stratification of mixed glial cells [35]. Primary microglia were then cultured in DMED-F12 medium that contained 2% serum overnight. Alexa Fluor 488-conjuaged Prx1 (F-Prx1, 30 nM) was added to the medium with or without 40 μM CD21 and incubated for 23.5 h. LysoTracker Red (a lysosome marker, 50 nM) and Hoechst 33342 were added and incubated for another 0.5 h. The phagocytosis of Prx1 by lysosomes was observed, and images were acquired using a fluorescent microscope (Nikon, Tokyo, Japan).

### Statistical analysis

The data are expressed as mean ± SD. The statistical analysis was performed using SPSS 26.0 software. Hemorrhagic volume in WT and MSR1^−/−^ mice was analyzed using two-way analysis of variance (ANOVA) followed by the Bonferroni-Holm post hoc test. The other data were analyzed using one-way ANOVA followed by the least significant difference post hoc test or Tamhane’s test for multi-group comparisons in WT mice. Values of *p* < 0.05 were considered statistically significant.

## Results

### Neuroprotective effect of CD21 in tPA-infused tMCAO mice

We first assessed the effects of CD21, tPA, and their combined treatment on neurovascular complications in tMCAO mice. Consistent with our previous report [26], CD21 (13.78 mg/kg) significantly improved neurobehavioral dysfunction and infarct volume (*p* < 0.01, vs. tMCAO group; Fig. 1c–f). CD21 significantly decreased hemoglobin content in the ischemic brain compared with the tMCAO group (*p* < 0.05; Fig. 1g, h). tPA (10 mg/kg) significantly increased hemoglobin content in the ischemic brain and aggravated neurological deficits (*p* < 0.01 and *p* < 0.05, respectively, vs. tMCAO group). Combined treatment with tPA and CD21 significantly attenuated ischemic brain injury and HT compared with the tPA-treated tMCAO group (*p* < 0.01). These results demonstrated the neuroprotective effect of CD21 against tPA-induced HT in tMCAO mice.

### Attenuation of BBB dysfunction by CD21 in tPA-infused tMCAO mice

**Fig. 2:**
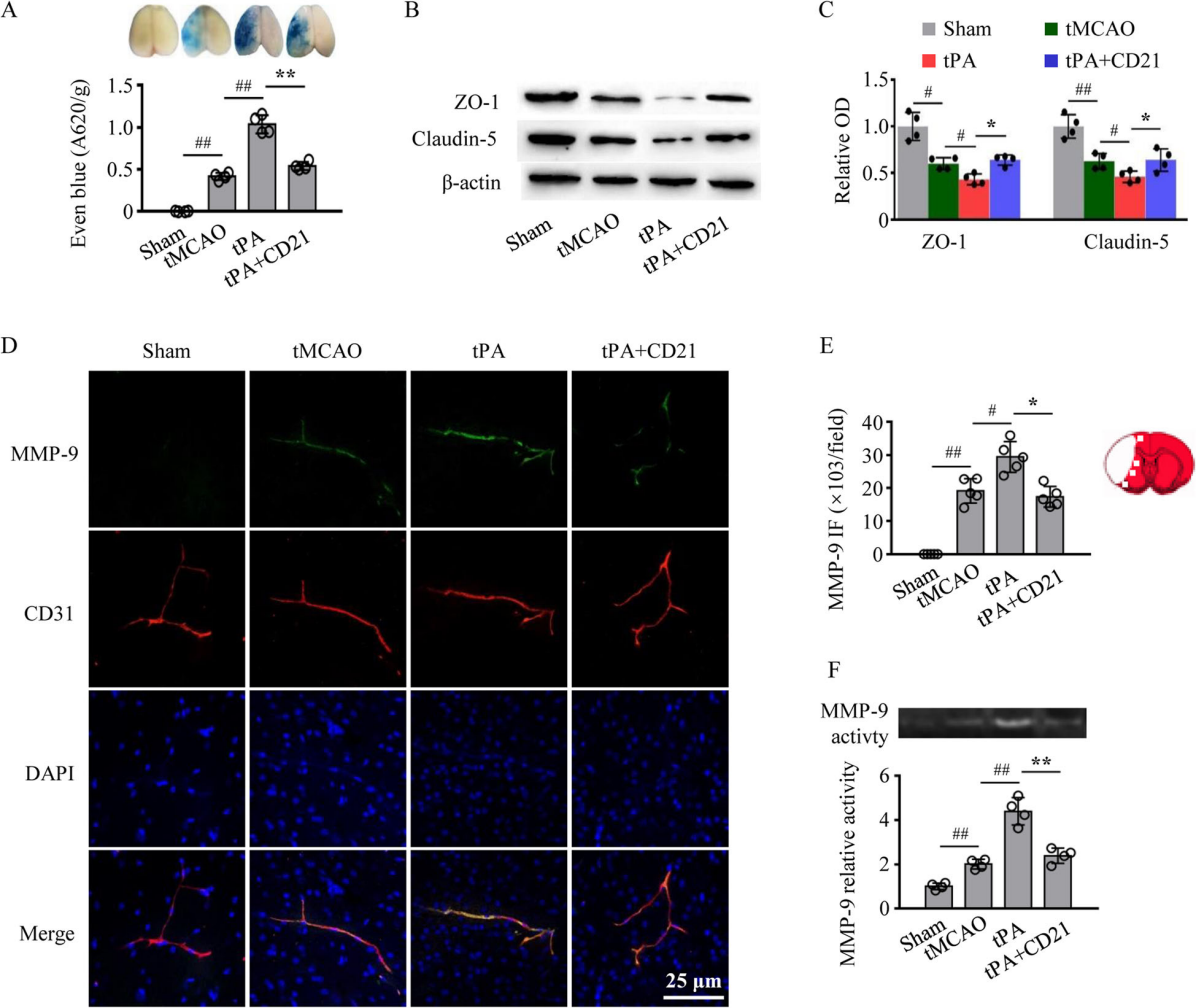
CD21 ameliorated blood-brain barrier disruption in tPA-infused tMCAO mice. **a** Representative images and statistical analysis of Evans blue extravasation in the ipsilateral brain hemisphere. **b**, **c** Representative immunoblots and quantitative analysis of tight junction proteins (ZO-1 and claudin-5) in the ipsilateral brain hemisphere. **d** Representative images of MMP-9 and CD31 double immunostaining surrounding cerebral vessels in the peri-infarct area. **e** Quantification of MMP-9 fluorescence intensity (FI) surrounding cerebral vessels in the peri-infarct area. **f** Representative zymography and quantification of MMP-9 activity in the ipsilateral brain hemisphere. The data are expressed as mean ± SD (*n* = 4/group in **a–c** and **f**, *n* = 5/group in **d** and **e**). ^#^*p* < 0.05, ^##^*p* < 0.01, vs. tMCAO group; *p < 0.05, **p < 0.01, vs. tPA group

Blood-brain barrier damage plays a vital role in HT after ischemic stroke. We examined the effect of CD21 on BBB integrity in tPA-infused tMCAO mice. As shown in Fig. 2a–c, tPA exacerbated Evans blue extravasation and the degradation of tight junction proteins (i.e., zonula occludens-1 [ZO-1] and claudin-5) in the ischemic brain (*p* < 0.01 and *p* < 0.05, respectively, vs. tMCAO group), which was significantly improved by combined treatment with tPA and CD21 (*p* < 0.01 and *p* < 0.05, respectively, *vs*. tPA group). We also found that tPA upregulated MMP-9 immunoreactivity and activity in the ischemic brain (*p* < 0.05 and *p* < 0.01, respectively, vs. tMCAO group), which was inhibited by combined treatment with tPA and CD21 (*p* < 0.05 and *p* < 0.01, respectively, vs. tPA group; Fig. 2d–f). Collectively, these results suggest that neuroprotection that is conferred by CD21 is partially associated with improvements in BBB integrity in tPA-treated tMCAO mice.

### Inhibition of neuroinflammation and the TLR4/NF-κB pathway by CD21 in tPA-infused tMCAO mice

**Fig. 3:**
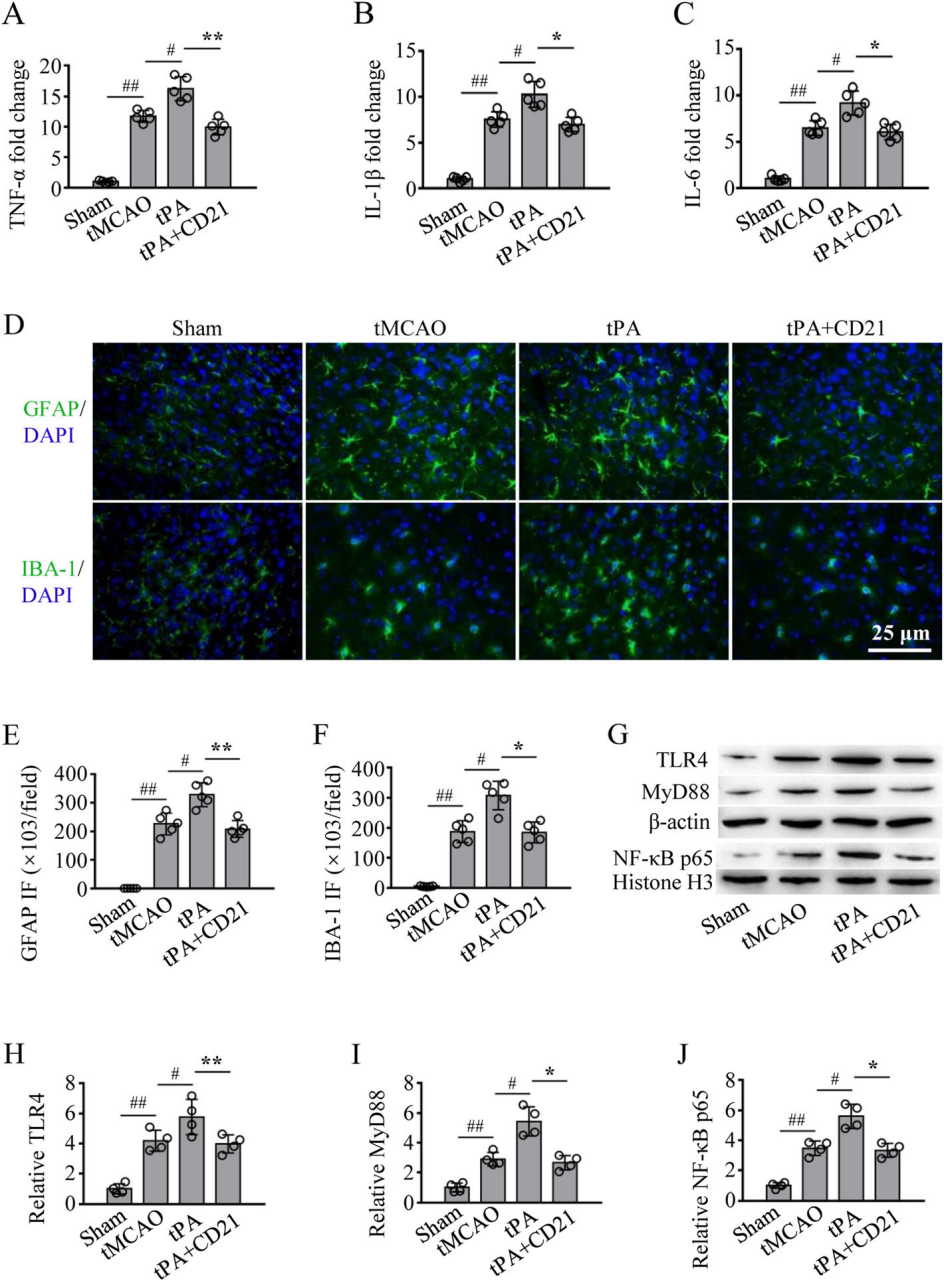
CD21 inhibited neuroinflammation and TLR4/NF-κB pathway activation in tPA-infused tMCAO mice. **a–c** Quantitative analysis of TNF-α, IL-1β, and IL-6 mRNA in the ipsilateral brain hemisphere, respectively. **d** Representative photomicrographs of GFAP-positive astrocytes and IBA-1-positive microglia/macrophages in the peri-infarct area in the ischemic brain. **e-f** Quantification of the fluorescence intensity (FI) of GFAP and IBA-1 in the peri-infarct area, respectively. **g** Representative immunoblots of TLR4/NF-κB signaling in the ipsilateral brain hemisphere. **h**, **i** Quantitative analysis of ratios of TRL4 and MyD88 to β-actin, respectively. **j** Quantitative analysis of the nuclear translocation ratio of NF-κB p65 to histone H3. The data are expressed as mean ± SD (*n* = 5/group in A-F, *n* = 4/group in **g–j**)*.* ^#^*p* < 0.05, ^##^*p* < 0.01, vs. tMCAO group; *p < 0.05, **p < 0.01, vs. tPA group

Neuroinflammation contributes to BBB dysfunction and HT after ischemic stroke. We investigated the effect of CD21 on neuroinflammation in tPA-infused tMCAO mice. As shown in Fig. 3a–c, tPA significantly upregulated the transcription of tumor necrosis factor-α (TNF-α), interleukin-1β (IL-1β), and IL-6 in the ipsilateral brain hemisphere compared with the tMCAO group, respectively (*p* < 0.05). Combined treatment with tPA and CD21 significantly downregulated the transcription of cytokines (*p* < 0.01 and *p* < 0.05, vs. tPA group). Similarly, tPA significantly increased the activation of astrocytes and microglia/macrophages in the peri-infarct area (*p* < 0.05, vs. tMCAO group). Combined treatment with tPA and CD21 significantly reduced the activation of both cell types (*p* < 0.01 and *p* < 0.05, respectively, vs. tPA group; Fig. 3d–f). Additionally, tPA significantly activated the post-ischemic TLR4/NF-κB signaling pathway, including increases in the expression of TLR4 and its downstream signaling factor MyD88 and nuclear translocation of NF-κB p65 (*p* < 0.05, vs. tMCAO group). Combined treatment with tPA and CD21 significantly reduced activation of the TLR4/NF-κB signaling pathway (*p* < 0.01 and *p* < 0.05, vs. tPA group; Fig. 3g–j).

### Downregulation of extracellular Prx1 levels by CD21 in tPA-infused tMCAO mice

**Fig. 4:**
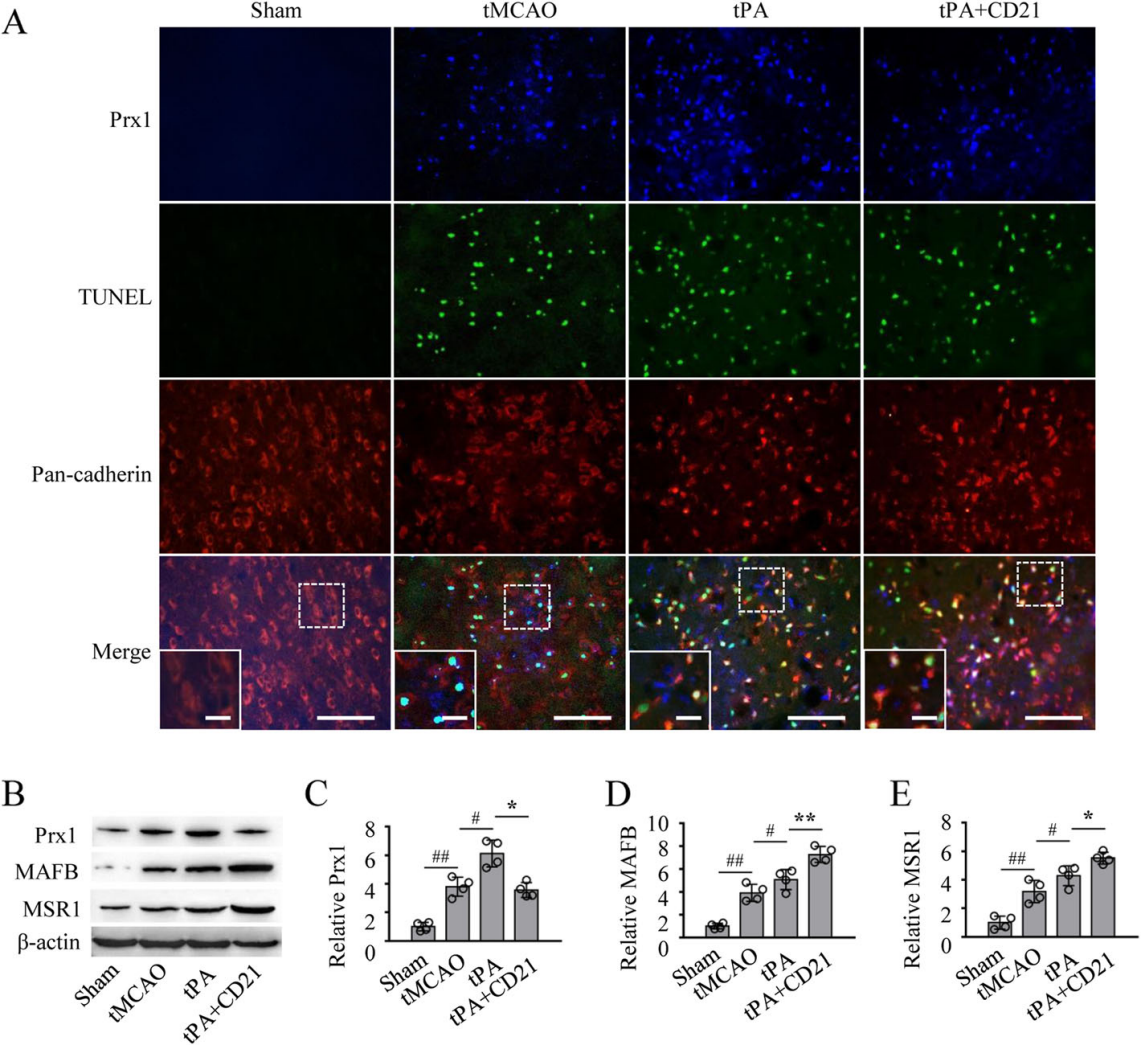
CD21 decreased extracellular Prx1 in the ischemic brain in tPA-infused tMCAO mice. **a** Representative photomicrographs of Prx1 immunoreactivity, TUNEL-positive apoptotic cells, and pan-cadherin-positive cell membrane in the peri-infarct area. **b–e** Representative Western blots and quantitative analysis of Prx1, MAFB, and MSR1 expression in the ipsilateral brain hemisphere. Scalebar (**a**) = 25 μm. The data are expressed as mean ± SD (*n* = 4/group). ^#^*p* < 0.05, ^##^*p* < 0.01, vs. tMCAO group; *p < 0.05, **p < 0.01, vs. tPA group

Extracellular Prx1 is an endogenous ligand of TLR4, which has been shown to be upregulated and detected around TUNEL-positive cells in mouse models of stroke [11, 16]. Western blot revealed that tPA significantly upregulated Prx1 levels in the ischemic brain hemisphere (*p* < 0.05, vs. tMCAO group), which was significantly inhibited by combined treatment with tPA and CD21 (*p* < 0.05, vs. tPA group; Fig. 4b, c). The immunofluorescent staining results showed that Prx1 outside the cell membrane (pan-cadherin-positive) of TUNEL-positive cells was upregulated in the peri-infarct area in the tPA group and downregulated by combined treatment with tPA and CD21 (Fig. 4a), indicating that combined treatment with tPA and CD21 reduced extracellular Prx1 from injured-to-dead brain cells.

Next, we examined the expression of V-maf musculoaponeurotic fibrosarcoma oncogene homolog B (MAFB) and MSR1 in the ipsilateral brain hemisphere, the function of which is to clear DAMPs (e.g., Prx1) after stroke [18, 19]. Their expression was significantly upregulated by tPA after ischemia (*p* < 0.05, vs. tMCAO group). Combined treatment with tPA and CD21 further significantly increased the expression of MAFB and MSR1 (*p* < 0.01 and *p* < 0.05, respectively, vs. tPA group; Fig. 4d, e).

### Role of MSR1 in CD21-induced Prx1 phagocytosis and protection against tPA-induced HT

**Fig. 5:**
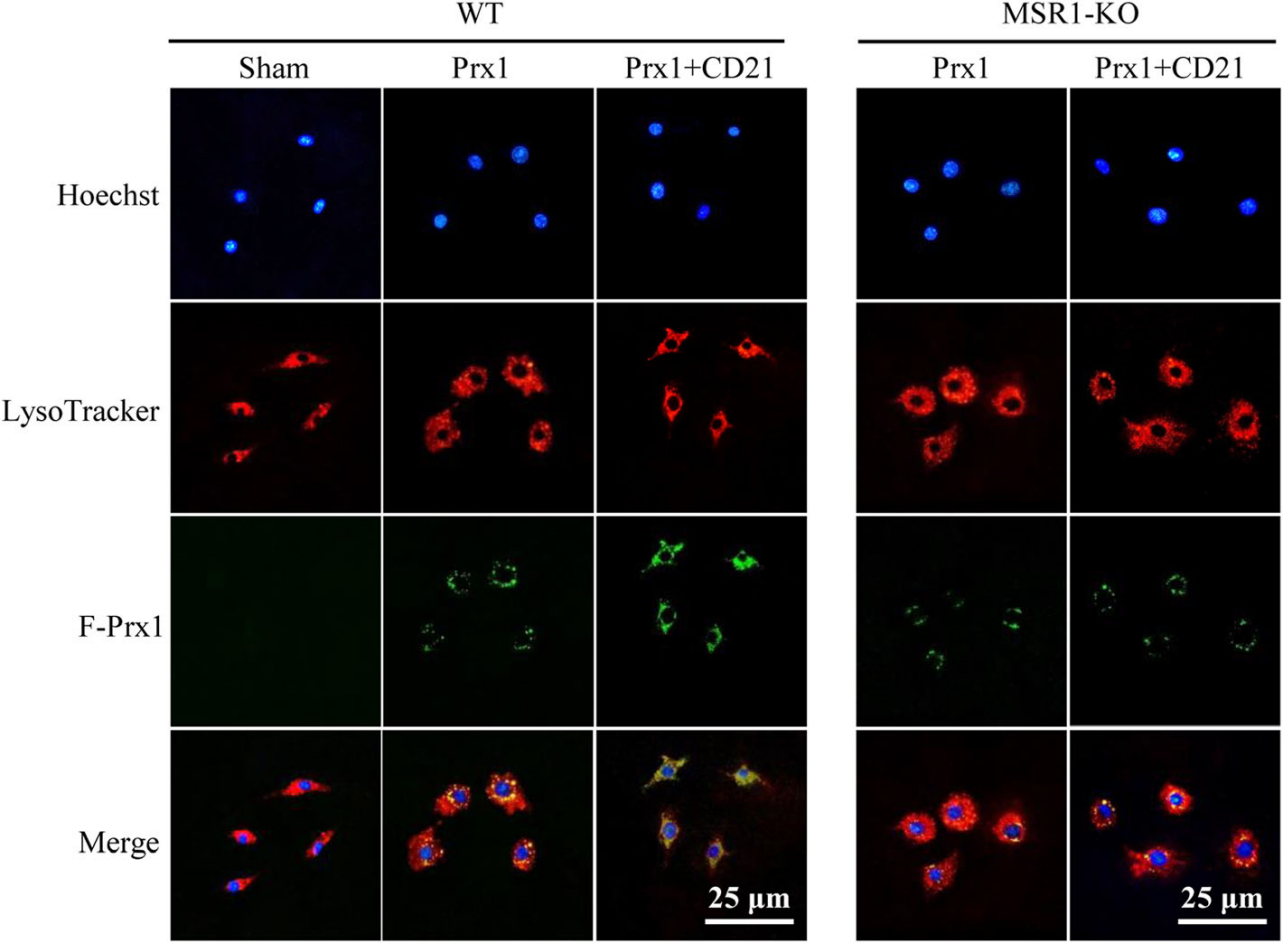
MSR1 knockout downregulated CD21-induced Prx1 phagocytosis in primary microglial cells. Representative photomicrographs of the localization of fluorescently labeled Prx1(F-Prx1) and lysosomes in primary cultured microglial cells from wildtype (WT) and MSR1^−/−^ (KO) mice

**Fig. 6:**
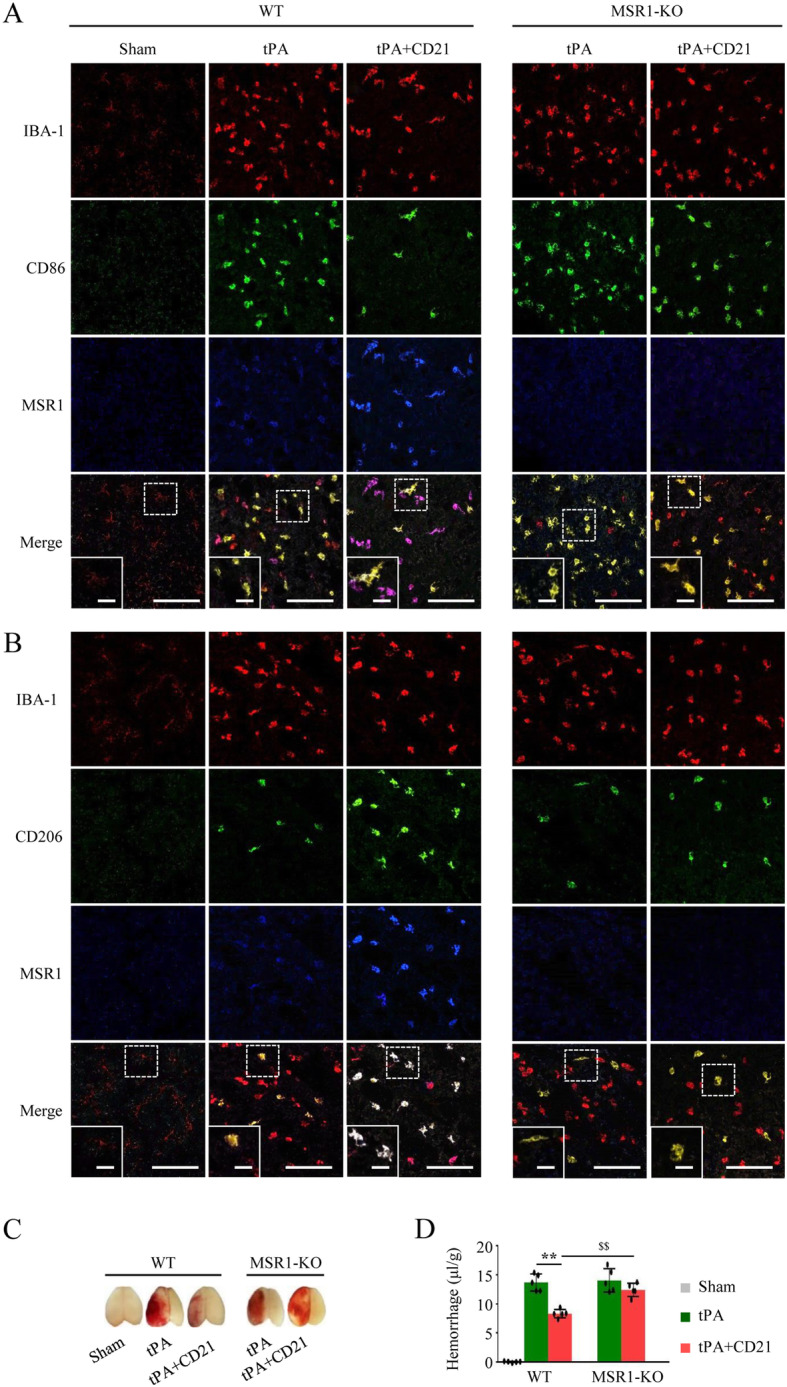
MSR1 knockout reduced CD21-induced protection against HT in tPA-infused tMCAO mice. **a** Representative immunofluorescent co-staining images of IBA-1, CD86, and MSR1 around the brain injury area. **b** Representative immunofluorescent co-staining images of IBA-1, CD206, and MSR1 around the brain injury area. **c** Representative images of cerebral hemorrhage 72 h after tMCAO in wildtype (WT) and MSR1^−/−^ (KO) mice. **d** Quantification of cerebral hemorrhagic volume in each group. Scalebar (**a**, **b**) = 50 μm. The data are expressed as mean ± SD (*n* = 5/group). **p < 0.01, vs. tPA group in WT mice; ^$$^*p* < 0.01, vs. tPA + CD21 group in WT mice

We further investigated the role of MSR1 in CD21-induced Prx1 clearance and protection against tPA-induced HT in in vitro cultured primary microglia and MSR1^−/−^ mice, respectively. As shown in Fig. 5, the immunostaining results showed that green fluorescently labeled Prx1 was co-stained with LysoTracker Red, indicating that extracellular Prx1 may be internalized by microglia for lysosomal degradation, resulting in Prx1 clearance. CD21 promoted Prx1 clearance in WT microglial cells, which was significantly inhibited in MSR1^−/−^ microglia. Additionally, CD21 promoted the expression of MSR1 and M2 polarization of microglia/macrophages in WT mice, and the effect of promoting MSR1 expression in M2-type cells was better than in M1-type cells. However, CD21 had little effect on the polarization of microglia/macrophages in MSR1^−/−^ mice (Fig. 6a, b). Moreover, CD21 significantly decreased hemoglobin levels in the ischemic brain after tPA infusion in WT mice but not in MSR1^−/−^ mice (*p* < 0.01, vs. tPA group; Fig. 6c, d). The protective effect of combined treatment with tPA and CD21 was weakened by MSR1 deficiency in MSR1^−/−^ mice vs. WT mice (*p* < 0.01). Collectively, these findings indicated that CD21 alleviated tPA-induced HT by accelerating MSR1-mediated Prx1 clearance in tMCAO mice.

## Discussion

Accumulating studies have focused on reducing HT by applying combined treatment with tPA and other neuroprotectants in acute ischemic stroke [36–38]. Previous studies demonstrated the neuroprotective effects of natural phthalides, such as ligustilide and butylphthalide, against ischemic and hemorrhagic stroke outcomes in ischemic and hemorrhagic animals [14, 39, 40]. As a newly synthesized phthalide derivative, the anti-neuroinflammatory and neuroprotective effects of CD21 have been investigated in different rodent models of ischemic stroke. Our previous studies showed that CD21 might alleviate post-ischemia inflammation and brain injury in tMCAO rats and mice [19, 26, 27]. In the present study, we further explored the effect of CD21 on tPA-induced HT in tMCAO mice. Combined treatment with tPA and CD21 significantly rescued tPA-mediated body weight loss in tMCAO mice (Supplementary Figure S3). Before the onset of brain ischemia, no difference in body weight was found among groups (*p* > 0.05). Three days after ischemic stroke, body weight in the tMCAO group significantly decreased (*p* < 0.01, vs. sham group). Compared with the tMCAO group, treatment with CD21 significantly ameliorated body weight loss (*p* < 0.05), whereas tPA worsened body weight loss (*p* < 0.05). Combined treatment with tPA and CD21 significantly rescued tPA-induced body weight loss in tMCAO mice (*p* < 0.01). We found that CD21 protected against tPA-induced HT in tMCAO mice, reflected by improvements in cerebral hemorrhage and injury, BBB disruption, cytokine transcription, glial cell activation, MAFB/MSR1-mediated Prx1 clearance, and TLR4/NF-κB pathway inhibition. Moreover, MSR1 deficiency significantly attenuated CD21-induced Prx1 phagocytosis in cultured microglial cells and tPA-induced HT in tMCAO mice.

Numerous studies reported that BBB damage is an important pathological process that is associated with thrombolytic hemorrhage in acute ischemic stroke [41]. In the present study, we first examined the effect of CD21 on BBB integrity and function in tPA-infused tMCAO mice. Our results showed that tPA significantly aggravated Evans blue extravasation and the degradation of tight junction proteins (i.e., ZO-1 and claudin-5) in tMCAO mice, which was effectively improved by CD21 treatment. TLR4 is involved in hemorrhagic transformation and BBB damage that are induced by delayed tPA administration, likely by increasing MMP-9 expression, in rats [8]. Ischemic stress-induced DAMP/TLR4/NF-κB pathway activation upregulates the expression and activation of MMPs. MMP-9 can strongly degrade tight junction proteins and the basement membrane, thereby aggravating BBB damage and the propensity for HT that is associated with ischemic brain injury and thrombolytic therapy [42]. The MMP-9 inhibitor BB-94 effectively decreased tPA-induced hemorrhagic volume and rate in cerebral ischemic animals [43, 44]. Therefore, MMP-9 expression has high predictive value for tPA-induced HT after acute ischemic stroke. In the present study, CD21 treatment significantly inhibited MMP-9 expression and activity in tPA-infused tMCAO mice. These findings suggest that the protective effect of CD21 against tPA-induced HT is associated with the attenuation of MMP-9-related BBB leakage and breakdown in acute ischemic stroke.

Previous studies reported that tPA-induced inflammation plays an important role in the progression of HT in acute ischemic stroke [7, 36, 45]. Potential treatment strategies that suppress tPA-related inflammation would likely improve patient outcomes. To explore the neuroinflammatory response in tMCAO rats, qRT-PCR is usually performed to examine mRNA levels of proinflammatory cytokines in ischemic brain tissues [14]. In the present study, we examined mRNA levels of proinflammatory cytokines in the ipsilateral hemisphere. Intrinsic glial cells and infiltrating leukocytes are the main immune cells that are involved in the neuroinflammatory response and neuronal injury after ischemic stroke [7]. Astrocytes are an important type of glial cell in regulating cerebral homeostasis and promoting glial regeneration [46, 47]. Microglia are resting mononuclear phagocytes in the healthy brain. They are sensitive to environmental changes and counteract threats to tissue integrity [45, 48, 49]. Once activated after stroke, both astrocytes and microglia release numerous cytokines that magnify inflammatory injury [46]. Blood-derived monocytes also infiltrate the brain and differentiate into macrophages following post-stroke BBB breakdown, which contributes to neuroinflammatory injury similarly to activated microglia in the acute phase of stroke [50]. Activated microglia/macrophages reach peak values on day 3 post-stroke and achieve a proportion of approximately 70% of all immune cells that participate in the neuroinflammatory response [51]. IBA-1-positive microglia/macrophages are generally positively associated with the neuroinflammatory response in stroke. Our previous studies found CD21-induced decreases in IBA-1-positive cells in ischemic brain tissues in tMCAO rats and 2VO mice, suggesting an anti-neuroinflammatory effect of CD21 in acute brain ischemia [19, 27]. In the present study, we observed elevations of mRNA levels of proinflammatory cytokines (i.e., TNF-α, IL-1β, and IL-6) in the ipsilateral hemisphere and the stronger activation of astrocytes and microglia/macrophages in the ischemic brain in tPA-infused tMCAO mice, which were significantly inhibited by combined treatment with tPA and CD21, suggesting that CD21 inhibited the tPA-induced neuroinflammatory response after ischemic stroke.

Toll-like receptors are a class of highly conserved membrane receptors in almost all cell types, and they are especially abundant in immune cells [52]. TLR4 has been identified as the major isoform of TLRs that trigger sterile inflammation in many central nervous system diseases [53]. Studies have demonstrated the direct upregulation of TLR4 expression and activation by DAMPs. For example, TLR4 expression in RAW264.7 cells was increased by exogenous Prx1, whereas TLR4 expression in the murine brain were decreased by Pxr1 knockout [54, 55]. Experimental and clinical studies have demonstrated the role of DAMPs (e.g., HMGB1 and Prxs) in TLR4-activated neuroinflammation after brain ischemia and hemorrhage [11, 16, 56, 57]. HMGB1 levels significantly increased in tPA-treated ischemic stroke patients and tMCAO rats [21, 22]. To our knowledge, only two recently published papers reported preventive effects of two compounds (glycyrrhizin and YC-1) against tPA-induced HT via the suppression of HMGB1/TLR2 or HMGB1/TLR4 signaling in ischemic stroke rats [21, 22]. In the present study, we detected Prx1/TLR4 pathway activation in tPA-infused tMCAO mice. Our Western blot and immunostaining results showed that tPA increased DAMP (Prx1) and TLR4/NF-κB activation in ischemic brain tissues in tMCAO mice, which was ameliorated by combined treatment with tPA and CD21 (Figs. 3g–j, 4a–c).

MSR1 has been shown to promote post-ischemic DAMP (HMGB1 and Prxs) clearance, leading to the resolution of neuroinflammation and attenuation of ischemic brain injury in mice and rats [18, 19]. DAMP internalization was reported to be largely mediated by MSR1 in a transcription factor (MAFB)-dependent manner in vitro and in tMCAO mice, whereas MAFB deficiency impaired the clearance of DAMPs and resulted in more severe inflammation and the exacerbation of neuronal injury in tMCAO mice [18]. Our previous study reported that the neuroprotective effect of CD21 (a novel synthesized neuroprotectant) was associated with the upregulation of MSR1-promoted Prx1 clearance and inhibition of neuroinflammation in tMCAO rats [19]. Emerging evidence suggests that efferocytosis or the phagocytic clearance of dead/dying cells by brain-resident microglia and infiltrating macrophages may reduce the release of DAMPs and promote the resolution of inflammation and restoration of brain homeostasis after stroke [58]. The present study mainly explored the effect of CD21 on the MSR1-mediated clearance of DAMPs (Prx1) in a mouse model of thrombolysis-induced HT. We found that CD21 significantly increased MSR1 expression in ischemic brain tissues in tPA-infused tMCAO mice in vivo (Fig. 4b) and Prx1 internalization via MSR1 in cultured primary microglia in vitro (Fig. 5). These results indicated that CD21 improved DAMP (Prx1) clearance, TLR4 pathway inhibition, and neuroinflammation resolution in ischemic brain tissues of tPA-infused tMCAO mice. However, unknown is whether MSR1 may accelerate the resolution of inflammation through direct induction of the phagocytosis of dead/dying cells (e.g., apoptotic neurons) after stroke. This possibility needs to be explored in future studies.

**Fig. 7:**
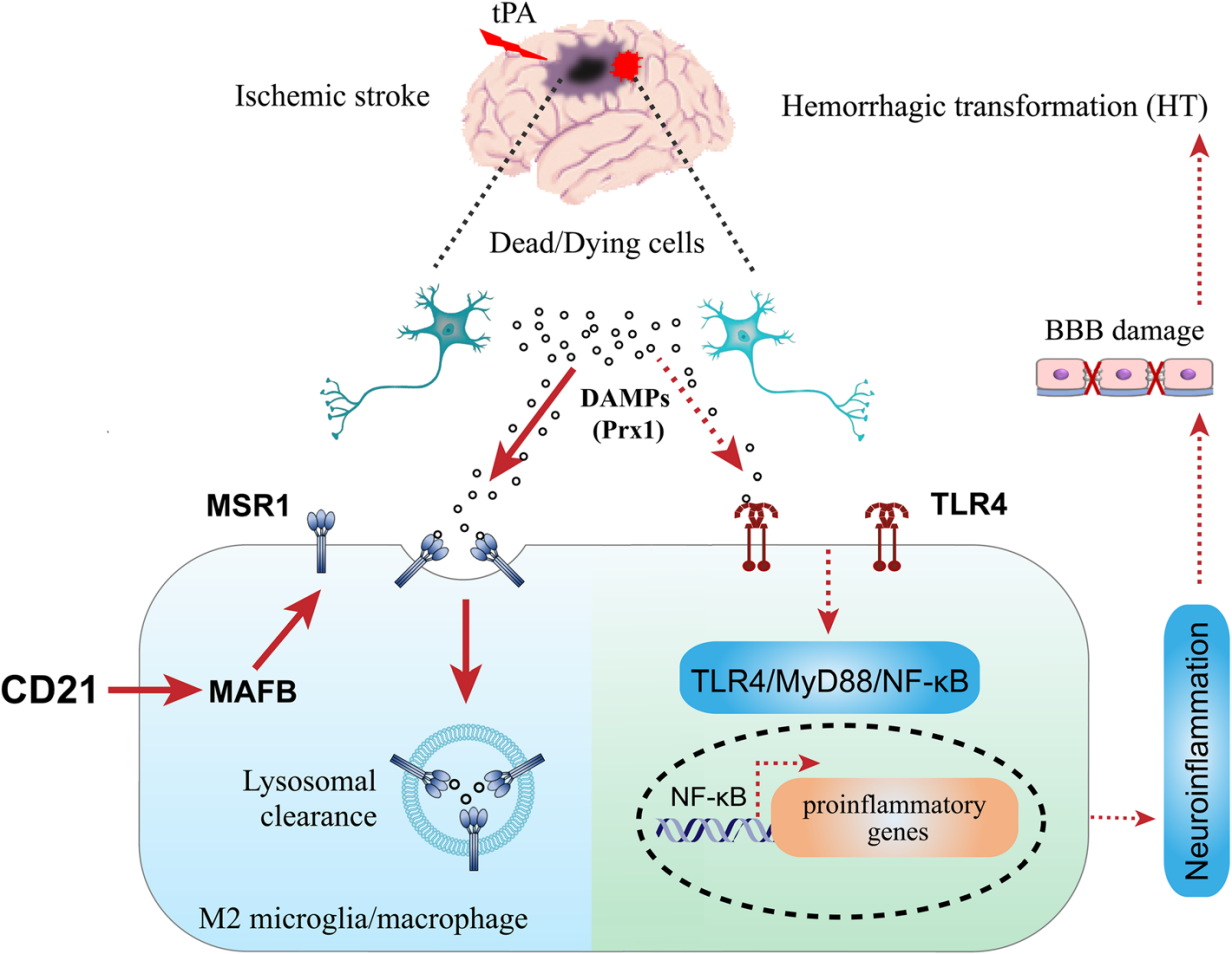
Schematic representation of the protective effect of CD21 against tPA-induced HT in acute ischemic stroke. CD21 increased MSR1 expression in M2 microglia/macrophages and MSR1-related lysosomal clearance of the DAMP Prx1, thereby ameliorating tPA-induced HT through inhibition of the DAMP (Prx1)/TLR4/NF-κB pathway and neuroinflammation and attenuation of BBB dysfunction in acute ischemic stroke

It has been demonstrated that MSR1 is predominantly expressed in cerebral microglia and macrophages in other tissues [59–61]. Previous studies showed that the loss of bone marrow cell-derived MSR1 exacerbated ischemic injury [18], highlighting the critical role of macrophage/monocyte-derived MSR1. The present findings suggest that CD21 possibly exerts its neuroprotective effect by acting on MSR1 that is expressed on IBA-1-positive microglia/macrophages. MSR1 is ablated in both microglia and macrophages/monocytes in global MSR1^−/−^ mice [20]. Therefore, notable is the role of MSR1 in the neuroprotective effect of CD21 against thrombolysis-related HT after acute ischemic stroke in global MSR1^−/−^ mice. However, we cannot exclude the possibility that MSR1 on tissue-resident macrophages in other organs may also indirectly contribute to a beneficial effect of CD21. This possibility will be explored in future studies. Numerous studies have shown that MSR1 that is expressed in M1- and M2-type microglia/macrophages exerts proinflammatory and anti-inflammatory effects, respectively [62–64]. In the M1 phenotype, MSR1 may act as a co-receptor along with TLRs for DAMPs and stimulate the production of inflammatory mediators, whereas MSR1 in the M2 phenotype can internalize DAMPs and induce DAMPs/TLR4/NF-κB pathway inhibition and inflammation resolution. Our previous study reported that CD21 increased the M2-polarization of microglia/macrophages in the brain in tMCAO mice and M2-polarization of BV2 microglia in vitro [26]. In the present study, we further examined the effect of CD21 on MSR1 immunoreactivity in M1 and M2 phenotypes in the brain in tPA-infused tMCAO mice (Fig. 6a, b). Shichita et al. reported that the infarct size in the brain in MSR1-deficient mice was larger than in WT mice [18]. Based on the previously reported neuroprotection that is conferred by CD21 against brain ischemia [19, 26, 27], our study further explored its effect on tPA-induced HT in acute ischemic stroke. We found that MSR1 knockout significantly attenuated the protective effect of CD21 against tPA-induced HT after tMCAO (Fig. 6c, d). Altogether, our findings suggest that CD21 inhibits the neuroinflammatory response through the induction M2-type anti-inflammatory microglia/macrophages, leading to the upregulation of MSR1 expression and DAMP (Prx1) clearance and inhibition of the TLR4/NF-κB pathway in tPA-treated tMCAO mice (Fig. 7).

## Conclusions

We found that CD21, a newly synthesized neuroprotectant, ameliorated tPA-induced HT in brain ischemia by accelerating Prx1 clearance in an MSR1-dependent manner. These findings suggest a novel synergistic strategy for the prevention of HT during thrombolysis in acute ischemic stroke through the enhancement of MSR1-mediated DAMP clearance.

## Supplementary Information

**Additional file 1: Supplemental Figure S1.** Chemical structure of Butylphthalide (A), Ligustilide (B) and CD21 (C). The molecular weight of both natural phthalides (Butylphthalide and Ligustilide) is 190, and the molecular weight of synthesized phthalide derivative CD21 is 262. **Supplemental Figure S2.** CD21 purity determination by Ultra high-performance liquid chromatography (UHPLC) analysis. Chromatographic separation was performed on Waters ACQUITY system and BEH C18 column (50 × 2.1 mm, 1.7 μm). The mobile phase was a mixture of acetonitrile and 0.1% phosphoric acid (30:70, v/v). The flow rate was 0.4 mL/min, and the column temperature was 35 °C. The detection wavelength was set at 225 nm. **Supplemental Figure S3.** Body weight loss of mice in the different groups. The data are expressed as mean ± SD, and analyzed by two way ANOVA with Bonferroni-Holm test (*n* = 10/group). ^#^*p* < 0.05, ^##^*p* < 0.01, *vs*. tMCAO group 3 days after stroke; **p < 0.01, *vs*. tPA group 3 days after stroke; ^$$^*p* < 0.01, *vs*. each group before stroke.

## Data Availability

The authors declare that they will provide all data and materials from this study upon reasonable request.

## Abbreviations

BBB: Blood-brain barrier
DAMP: Damage-associated molecular pattern
ECA: External carotid artery
F-Prx1: Alexa Fluor 488-conjuaged Prx1
FI: Fluorescence intensity
HT: Hemorrhagic transformation
ICA: Internal carotid artery
ICH: Intracerebral hemorrhage
IL-1β: Interleukin-1β
IL-6: Interleukin-6
MAFB: V-maf musculoaponeurotic fibrosarcoma oncogene homolog B
MCA: Middle cerebral artery
MMP-9: Matrix metalloproteinase 9
mNSS: Modified neurological severity score
MSR1: Macrophage scavenger receptor 1
MSR1^−/−^: MSR1 knockout
MyD88: Myeloid differentiation factor 88
NF-κB: Nuclear factor κB
OD: Optical density
Prx1: Peroxiredoxin 1
qPCR: Quantitative polymerase chain reaction
TLR4: Toll-like receptor 4
tMCAO: Transient middle cerebral artery occlusion
TNF-α: Tumor necrosis factor α
tPA: Tissue plasminogen activator
TTC: 2,3,5-Triphenyltetrazoliumchloride
WT: Wildtype
ZO-1: Zonula occludens-1

## References

[1] He Z, Ning N, Zhou Q, Khoshnam SE, Farzaneh M (2020). Mitochondria as a therapeutic target for ischemic stroke. Free Radic Biol Med..

[2] Mikulik R, Wahlgren N (2015). Treatment of acute stroke: an update. J Intern Med..

[3] Hacke W, Kaste M, Bluhmki E, Brozman M, Davalos A, Guidetti D (2008). Thrombolysis with alteplase 3 to 4.5 hours after acute ischemic stroke. N Engl J Med..

[4] Shi KB, Tian DC, Li ZG, Ducruet AF, Lawton MT, Shi FD (2019). Global brain inflammation in stroke. Lancet Neurol..

[5] Chaudhry SR, Hafez A, Jahromi BR, Kinfe TM, Lamprecht A, Niemelä M (2018). Role of damage associated molecular pattern molecules (DAMPs) in aneurysmal subarachnoid hemorrhage (aSAH). Int J Mol Sci..

[6] Zhou Y, Wang YC, Wang J, Stetler RA, Yang QW (2014). Inflammation in intracerebral hemorrhage: from mechanisms to clinical translation. Prog Neurobiol..

[7] Shi K, Zou M, Jia DM, Shi S, Yang X, Liu Q, Dong JF, Sheth KN, Wang X, Shi FD (2021). tPA mobilizes immune cells that exacerbate hemorrhagic tansformation in stroke. Circ Res..

[8] García-Culebras A, Palma-Tortosa S, Moraga A, García-Yébenes I, Durán-Laforet V, Cuartero MI, de la Parra J, Barrios-Muñoz AL, Díaz-Guzmán J, Pradillo JM, Moro MA, Lizasoain I (2017). Toll-like receptor 4 mediates hemorrhagic transformation after delayed tissue plasminogen activator administration in in situ thromboembolic stroke. Stroke..

[9] Iadecola C, Anrather J (2011). The immunology of stroke: from mechanisms to translation. Nat Med..

[10] Shichita T, Ito M, Yoshimura A (2014). Post-ischemic inflammation regulates neural damage and protection. Front Cell Neurosci..

[11] Shichita T, Hasegawa E, Kimura A, Morita R, Sakaguchi R, Takada I, Sekiya T, Ooboshi H, Kitazono T, Yanagawa T, Ishii T, Takahashi H, Mori S, Nishibori M, Kuroda K, Akira S, Miyake K, Yoshimura A (2012). Peroxiredoxin family proteins are key initiators of post-ischemic inflammation in the brain. Nat Med..

[12] Salzano S, Checconi P, Hanschmann EM, Lillig CH, Bowler LD, Chan P, Vaudry D, Mengozzi M, Coppo L, Sacre S, Atkuri KR, Sahaf B, Herzenberg LA, Herzenberg LA, Mullen L, Ghezzi P (2014). Linkage of inflammation and oxidative stress via release of glutathionylated peroxiredoxin-2, which acts as a danger signal. Proc Natl Acad Sci U S A..

[13] Lu Y, Zhang XS, Zhang ZH, Zhou XM, Gao YY, Liu GJ, Wang H, Wu LY, Li W, Hang CH (2018). Peroxiredoxin 2 activates microglia by interacting with Toll-like receptor 4 after subarachnoid hemorrhage. J Neuroinflammation..

[14] Kuang X, Wang LF, Yu L, Li YJ, Wang YN, He Q, Chen C, du JR (2014). Ligustilide ameliorates neuroinflammation and brain injury in focal cerebral ischemia/reperfusion rats: involvement of inhibition of TLR4/peroxiredoxin 6 signaling. Free Radic Biol Med..

[15] Mao XN, Zhou HJ, Yang XJ, Zhao LX, Kuang X, Chen C, Liu DL, du JR (2017). Neuroprotective effect of a novel gastrodin derivative against ischemic brain injury: involvement of peroxiredoxin and TLR4 signaling inhibition. Oncotarget..

[16] Liu DL, Zhao LX, Zhang S, Du JR (2016). Peroxiredoxin 1-mediated activation of TLR4/NF- TLR4/NF-κB pathway contributes to neuroinflammatory injury in intracerebral hemorrhage. Int Immunopharmacol..

[17] Shichita T, Ago T, Kamouchi M, Kitazono T, Yoshimura A, Ooboshi H (2012). Novel therapeutic strategies targeting innate immune responses and early inflammation after stroke. J Neurochem..

[18] Shichita T, Ito M, Morita R, Komai K, Noguchi Y, Ooboshi H, Koshida R, Takahashi S, Kodama T, Yoshimura A (2017). MAFB prevents excess inflammation after ischemic stroke by accelerating clearance of damage signals through MSR1. Nat Med..

[19] Zou X, Yang XJ, Gan YM, Liu DL, Chen C, Duan W, du JR (2020). Neuroprotective effect of phthalide derivative CD21 against ischemic brain injury: involvement of MSR1 mediated DAMP peroxiredoxin1 clearance and TLR4 signaling inhibition. J Neuroimmune Pharmacol..

[20] Zhao SJ, Kong FQ, Jie J, Li Q, Liu H, Xu AD, Yang YQ, Jiang B, Wang DD, Zhou ZQ, Tang PY, Chen J, Wang Q, Zhou Z, Chen Q, Yin GY, Zhang HW, Fan J (2020). Macrophage MSR1 promotes BMSC osteogenic differentiation and M2-like polarization by activating PI3K/AKT/GSK3β/β-catenin pathway. Theranostics..

[21] Chen H, Guan B, Wang B, Pu H, Bai X, Chen X, Liu J, Li C, Qiu J, Yang D, Liu K, Wang Q, Qi S, Shen J (2020). Glycyrrhizin prevents hemorrhagic transformation and improves neurological outcome in ischemic stroke with delayed thrombolysis through targeting peroxynitrite-mediated HMGB1 signaling. Transl Stroke Res..

[22] Kong L, Ma Y, Wang Z, Liu N, Ma G, Liu C, Shi R, du G (2021). Inhibition of hypoxia inducible factor 1 by YC-1 attenuates tissue plasminogen activator induced hemorrhagic transformation by suppressing HMGB1/TLR4/NF-κB mediated neutrophil infiltration in thromboembolic stroke rats. Int Immunopharmacol..

[23] Chen X, Wang K (2016). The fate of medications evaluated for ischemic stroke pharmacotherapy over the period 1995-2015. Acta Pharm Sin B..

[24] Kong LS, Liu LY, Zhao P, Hong B, Han XD, Ji XY (2020). Effects of butylphthalide on hemorrhagic transformation after intravenous thrombolysis of recombinant tissue plasminogen activator in acute cerebral infarction. Chin J New Drugs Clin Rem..

[25] Chen C, Du JR, Shi MQ, Miao XN, San N, Wang PH (2017). 3-Alkyl-5,6-dioxo-substituted phthalein compounds, and preparation method and use thereof.

[26] Gan YM, Liu DL, Chen C, Duan W, Yang YX, Du JR (2020). Phthalide derivative CD21 alleviates cerebral ischemia-induced neuroinflammation: involvement of microglial M2 polarization via AMPK activation. Eur J Pharmacol..

[27] Li X, Shi MQ, Chen C, Du JR (2020). Phthalide derivative CD21 ameliorates ischemic brain injury in a mouse model of global cerebral ischemia: involvement of inhibition of NLRP3. Int Immunopharmacol..

[28] Mao L, Li P, Zhu W, Cai W, Liu Z, Wang Y, Luo W, Stetler RA, Leak RK, Yu W, Gao Y, Chen J, Chen G, Hu X (2017). Regulatory T cells ameliorate tissue plasminogen activator-induced brain hemorrhage after stroke. Brain..

[29] Shi Y, Zhang L, Pu H, Mao L, Hu X, Jiang X, Xu N, Stetler RA, Zhang F, Liu X, Leak RK, Keep RF, Ji X, Chen J (2016). Rapid endothelial cytoskeletal reorganization enables early blood-brain barrier disruption and long-term ischaemic reperfusion brain injury. Nat Commun..

[30] Pan XW, Wang MG, Gong SS, Sun MH, Wang Y, Zhang YY (2020). YiQiFuMai lyophilized injection ameliorates tPA-induced hemorrhagic transformation by inhibiting cytoskeletal rearrangement associated with ROCK1 and NF-κB signaling pathways. J Ethnopharmacol..

[31] Chen J, Sanberg PR, Li Y, Wang L, Lu M, Willing AE, Sanchez-Ramos J, Chopp M (2001). Intravenous administration of human umbilical cord blood reduces behavioral deficits after stroke in rats. Stroke..

[32] Qiao Y, Ma Q, Zhai HF, Li Y, Tang MK (2019). Exposure to female estrous is beneficial for male mice against transient ischemic stroke. Neurol Res..

[33] Liu H, Wang Y, Xiao Y, Hua Z, Cheng J, Jia J (2016). Hydrogen sulfide attenuates tissue plasminogen activator-induced cerebral hemorrhage following experimental stroke. Transl Stroke Res..

[34] Zeng CY, Yang TT, Zhou HJ, Zhao Y, Kuang X, Duan W, du JR (2019). Lentiviral vector-mediated overexpression of Klotho in the brain improves Alzheimer’s disease-like pathology and cognitive deficits in mice. Neurobiol Aging..

[35] Zhang LQ, Zhang ZG, Xu Y (2015). A improved method for cultivating primary microglia of the rat. Chin J Neuroanat..

[36] Zhu Z, Fu Y, Tian D, Sun N, Han W, Chang G, Dong Y, Xu X, Liu Q, Huang DR, Shi FD (2015). Combination of the immune modulator fingolimod with alteplase in acute ischemic stroke: a pilot trial. Circulation..

[37] Li Y, Zhu ZY, Lu BW, Huang TT, Zhang YM, Zhou NY, Xuan W, Chen ZA, Wen DX, Yu WF, Li PY (2019). Rosiglitazone ameliorates tissue plasminogen activator-induced brain hemorrhage after stroke. CNS Neurosci Ther..

[38] Lu D, Liu Y, Mai H, Zang J, Shen L, Zhang Y, Xu A (2018). Rosuvastatin reduces neuroinflammation in the hemorrhagic transformation after rt-PA treatment in a mouse model of experimental stroke. Front Cell Neurosci..

[39] Chen H, He Y, Chen S, Qi S, Shen J (2020). Therapeutic targets of oxidative/nitrosative stress and neuroinflammation in ischemic stroke: applications for natural product efficacy with omics and systemic biology. Pharmacol Res..

[40] Zeng Z, Gong X, Hu Z (2020). L-3-n-butylphthalide attenuates inflammation response and brain edema in rat intracerebral hemorrhage model. Aging..

[41] Sifat AE, Vaidya B, Abbruscato TJ (2017). Blood-brain barrier protection as a therapeutic strategy for acute ischemic stroke. AAPS J..

[42] Ma G, Pan Z, Kong L, Du G (2021). Neuroinflammation in hemorrhagic transformation after tissue plasminogen activator thrombolysis: potential mechanisms, targets, therapeutic drugs and biomarkers. Int Immunopharmacol..

[43] Sumii T, Lo EH (2002). Involvement of matrix metalloproteinase in thrombolysis-associated hemorrhagic transformation after embolic focal ischemia in rats. Stroke..

[44] Lapchak PA, Chapman DF, Zivin JA (2000). Metalloproteinase inhibition reduces thrombolytic (tissue plasminogen activator)-induced hemorrhage after thromboembolic stroke. Stroke..

[45] Lenglet S, Montecucco F, Denes A, Coutts G, Pinteaux E, Mach F, Schaller K, Gasche Y, Copin JC (2014). Recombinant tissue plasminogen activator enhances microglial cell recruitment after stroke in mice. J Cereb Blood Flow Metab..

[46] Benveniste EN (1998). Cytokine actions in the central nervous system. Cytokine Growth Factor Rev..

[47] Liu Z, Chopp M (2016). Astrocytes, therapeutic targets for neuroprotection and neurorestoration in ischemic stroke. Prog Neurobiol..

[48] Taylor RA, Sansing LH (2013). Microglial responses after ischemic stroke and intracerebral hemorrhage. Clin Dev Immunol..

[49] Zhao SC, Ma LS, Chu ZH, Xu H, Wu WQ, Liu F (2017). Regulation of microglial activation in stroke. Acta Pharmacol Sin..

[50] Geissmann F, Manz MG, Jung S, Sieweke MH, Merad M, Ley K (2010). Development of monocytes, macrophages, and dendritic cells. Science..

[51] Gelderblom M, Leypoldt F, Steinbach K, Behrens D, Choe CU, Siler DA, Arumugam TV, Orthey E, Gerloff C, Tolosa E, Magnus T (2009). Temporal and spatial dynamics of cerebral immune cell accumulation in stroke. Stroke..

[52] Muzio M, Polentarutti N, Bosisio D, Prahladan MK, Mantovani A (2000). Toll-like receptors: a growing family of immune receptors that are differentially expressed and regulated by different leukocytes. J Leukoc Biol..

[53] Kong Y, Le YY (2011). Toll-like receptors in inflammation of the central nervous system. Int Immunopharmacol..

[54] Riddell JR, Wang XY, Minderman H, Gollnick SO (2010). Peroxiredoxin 1 stimulates secretion of proinflammatory cytokines by binding to TLR4. J Immunol..

[55] Liu Q, Zhang Y (2019). PRDX1 enhances cerebral ischemia-reperfusion injury through activation of TLR4-regulated inflammation and apoptosis. Biochem Biophys Res Commun..

[56] Heula AL, Ohlmeier S, Sajanti J, Majamaa K (2013). Characterization of chronic subdural hematoma fluid proteome. Neurosurgery..

[57] Zhou Y, Xiong KL, Lin S, Zhong Q, Lu FL, Liang H (2010). Elevation of high-mobility group protein box-1 in serum correlates with severity of acute intracerebral hemorrhage. Mediators Inflamm..

[58] Elliott MR, Koster KM, Murphy PS (2017). Efferocytosis signaling in the regulation of macrophage inflammatory responses. J Immunol..

[59] Christie RH, Freeman M, Hyman BT (1996). Expression of the macrophage scavenger receptor, a multifunctional lipoprotein receptor, in microglia associated with senile plaques in Alzheimer’s disease. Am J Pathol..

[60] Naito M, Kodama T, Matsumoto A, Doi T, Takahashi K (1991). Tissue distribution, intracellular localization, and in vitro expression of bovine macrophage scavenger receptors. Am J Pathol..

[61] Platt N, Gordon S (2001). Is the class A macrophage scavenger receptor (SR-A) multifunctional? The mouse’s tale. J Clin Invest..

[62] Komai K, Shichita T, Ito M, Kanamori M, Chikuma S, Yoshimura A (2017). Role of scavenger receptors as damage-associated molecular pattern receptors in Toll-like receptor activation. Int Immunol..

[63] Kumar R, Sharma A, Padwad Y, Sharma R (2020). Preadipocyte secretory factors differentially modulate murine macrophage functions during aging which are reversed by the application of phytochemical EGCG. Biogerontology..

[64] Canton J, Neculai D, Grinstein S (2013). Scavenger receptors in homeostasis and immunity. Nat Rev Immunol..

