# Supplementary material for: Phthalide derivative CD21 attenuates tissue plasminogen activator-induced hemorrhagic transformation in ischemic stroke by enhancing macrophage scavenger receptor 1-mediated DAMP (peroxiredoxin 1) clearance
Source: J Neuroinflammation. 2021 Jun 24;18:143. doi: 10.1186/s12974-021-02170-7 (PMC8223381; doi:10.1186/s12974-021-02170-7)
Supplement: Supplementary file 1 — Additional file 1: Supplemental Figure S1. Chemical structure of Butylphthalide (A), Ligustilide (B) and CD21 (C). The molecular weight of both natural phthalides (Butylphthalide and Ligustilide) is 190, and the molecular weight of synthesized phthalide derivative CD21 is 262. Supplemental Figure S2. CD21 purity determination by Ultra high-performance liquid chromatography (UHPLC) analysis. Chromatographic separation was performed on Waters ACQUITY system and BEH C18 column (50 × 2.1 mm, 1.7 μm). The mobile phase was a mixture of acetonitrile and 0.1% phosphoric acid (30:70, v/v). The flow rate was 0.4 mL/min, and the column temperature was 35 °C. The detection wavelength was set at 225 nm. Supplemental Figure S3. Body weight loss of mice in the different groups. The data are expressed as mean ± SD, and analyzed by two way ANOVA with Bonferroni-Holm test (n = 10/group). #p < 0.05, ##p < 0.01, vs. tMCAO group 3 days after stroke; **p < 0.01, vs. tPA group 3 days after stroke; $$p < 0.01, vs. each group before stroke. [file 12974_2021_2170_MOESM1_ESM.doc]

A B C

**
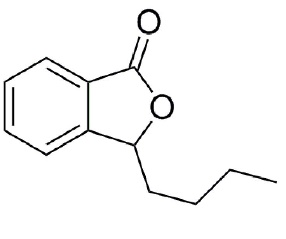

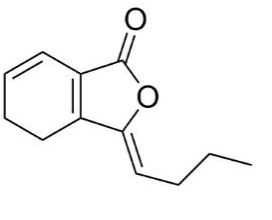
**
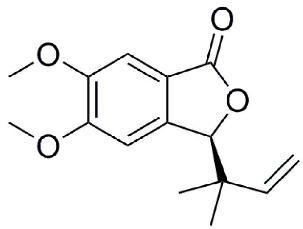


**Supplemental Figure S1.** Chemical structure of Butylphthalide (A), Ligustilide (B) and CD21 (C). The molecular weight of both natural phthalides (Butylphthalide and Ligustilide) is 190, and the molecular weight of synthesized phthalide derivative CD21 is 262.


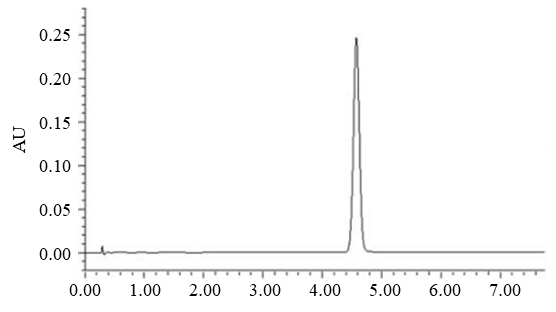


**Supplemental Figure S2.** CD21 purity determination by Ultra high-performance liquid chromatography (UHPLC) analysis**.** Chromatographic separation was performed on Waters ACQUITY system and BEH C18 column (50 × 2.1 mm, 1.7 μm). The mobile phase was a mixture of acetonitrile and 0.1% phosphoric acid (30:70, v/v). The flow rate was 0.4 mL/min, and the column temperature was 35 °C. The detection wavelength was set at 225 nm.


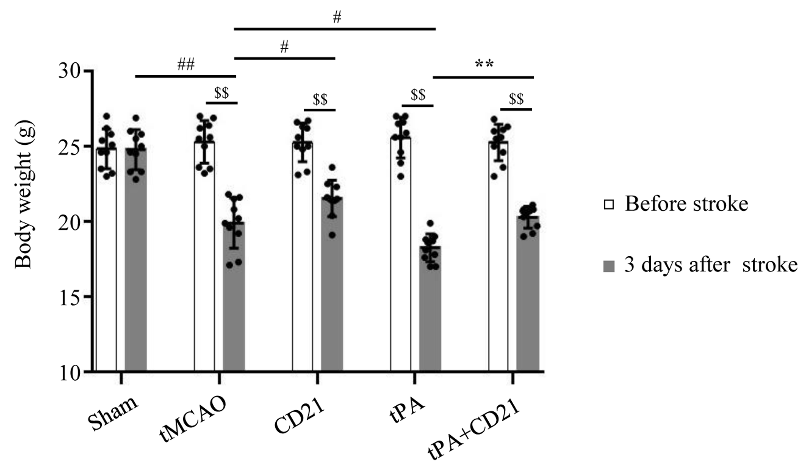


**Supplemental Figure S3. Body weight loss of mice in the different groups.** The data are expressed as mean ± SD, and analyzed by two way ANOVA with Bonferroni-Holm test (*n* = 10/group). ^#^*p* < 0.05, ^##^*p* < 0.01, *vs*. tMCAO group 3 days after stroke; *******p* < 0.01, *vs*. tPA group 3 days after stroke; ^$$^*p* < 0.01, *vs*. each group before stroke.
